# Modifiable and Non-Modifiable Risk Factors and Vascular Damage Progression in Type 2 Diabetes: A Primary Care Analysis

**DOI:** 10.3390/jcm14093155

**Published:** 2025-05-02

**Authors:** Carlo Fabris, Elena Rizzo, Stefano Bertolissi, Lucia Casatta, Massimo Pavan, Pierluigi Toniutto

**Affiliations:** 1 District of Udine, Azienda Sanitaria Universitaria Friuli Centrale, Piazzale S. Maria della Misericordia 1, 33100 Udine, Italy; 2 Department of Medicine, University of Udine, Piazzale S. Maria della Misericordia 1, 33100 Udine, Italy

**Keywords:** type 2 diabetes, vascular damage, microalbuminuria

## Abstract

**Background/Objectives:** Type 2 diabetes mellitus (DM2) is characterized by the development of micro/macro-vascular complications over time. Factors influencing their course may present specific features in the primary care context. This study aims to identify predictive factors for the evolution of micro/macro-vascular pathology in DM2 patients and evaluate interventions implemented by general practitioners (GPs) in this context. **Methods:** From the medical records of 1169 DM2 patients from 13 Italian GPs, demographic, socio-environmental, and clinical data were recorded, along with the presence and degree of arterial hypertension and components of diabetic micro/macroangiopathy at the time of study entry and 5 years prior. Laboratory parameters and therapies from the last three years were recorded. **Results:** Compared to 5 years prior, at the study entry, the number of patients presenting at least one micro- or macro-vascular complication increased from 192 (16.4%) to 344 (29.4%) and from 245 (21.0%) to 350 (29.9%). At the logistic regression, microalbuminuria determination appeared to be the strongest predictor of vascular damage progression, followed by decreasing LDL cholesterol values induced by lipid-lowering therapy. Male gender, age >75 years, and smoking history were associated with greater vascular damage progression in the ANOVA repeated measures test. **Conclusions:** Advanced age, male gender, and smoking history proved strongly associated with the presence and extent of damage progression. GPs appear to adopt a more aggressive approach in treating risk factors (particularly lipid profile) for damage progression in these patients. Microalbuminuria has proven to be by far the marker most strongly associated with vascular damage progression.

## 1. Introduction

Type two diabetes mellitus (DM2) certainly represents a condition associated with significant professional commitment for general practitioners (GPs). It is a frequent disease (about a hundred cases managed by a GP with 1500 patients), chronic (accompanying the person for the rest of their life from onset) [1], and frequently associated with particularly concerning complications, the latter constituting the most important challenge for GPs. These complications are represented by pathologies associated with diabetic microangiopathy (nephropathy, retinopathy, peripheral neuropathy) and macroangiopathy (ischemic heart, peripheral vascular, and cerebral vascular disease, abdominal aortic aneurysm) [2,3]. Currently, maintaining sufficient glycemic control while avoiding severe hyper and hypoglycemia has become a professional task that is easily accomplished in most primary care settings. However, avoiding or at least significantly slowing the progression of vascular damage, both micro- and macroangiopathy, are extraordinarily more difficult objectives to achieve.

Numerous studies are present in the literature regarding the progression of both micro- and macro-vascular damage in patients with DM2 [4,5]. Studies over the years have followed and chased each other in proposing evolutionary models, diagnostic–therapeutic algorithms, and future-oriented intervention strategies [6,7,8]. The field is extraordinarily complex as we must consider non-modifiable variables (genetics/age/sex) [9,10], environmental variables such as diet type, socioeconomic context (education/economic security/family composition) [11], and even important negative habits such as smoking and alcohol consumption and positive ones such as the presence and extent of physical activity [12]. The progression of vascular damage in DM2 could also be influenced by the concomitant presence of other pathologies linked to diabetic disease and associated with it, such as arterial hypertension and other cardiovascular diseases [13]. Although less known, other diseases, such as those affecting the central and peripheral nervous system, could play a role. With increasing survival, a new cohort of very elderly diabetic patients, strongly co-morbid, is advancing powerfully, for whom disease evolution due to interaction with other age-related conditions is certainly not well known.

For their part, GPs have valid and simply obtainable diagnostic tools, historically aimed at controlling diabetic disease and evaluating kidney functional status. These are glycated hemoglobin determination, serum creatinine, and microalbuminuria. These parameters are useful in establishing the degree of control of diabetic disease. More debatable is if the progression of vascular damage associated with DM2 could be extrapolated from the combination of their results [14]. Contextually, there is a strong association between LDL cholesterol levels and the onset and evolution of diabetic vascular disease, particularly macroangiopathy. Hence, from an operational point of view, the indication has emerged to decrease LDL values below 100 mg/dL in these patients and below 70 mg/dL in the presence of cardiovascular damage [15]. The European Society of Cardiology has produced a specific application for patients with DM2 aged between 40 and 70 years without previous significant vascular events [16]. The use of this application should help physicians in managing diabetic patients and preventing a more unfavorable disease evolution. However, studies focused on determining the evolutionary risk are almost exclusively performed by diabetologists. In the primary care context, studies have almost always been limited to a snapshot of reality rather than an attempt to interpret it.

This work, conducted entirely within general medicine, aimed to a) analyze the presence and predictive factors of micro/macro-vascular pathology evolution in DM2 considering demographic, socio-environmental, and clinical variables and b) evaluate interventions implemented by GPs, both diagnostic and therapeutic, in relation to the risk and actual progression of diabetic micro/macroangiopathy.

## 2. Materials and Methods

Thirteen GPs from the Udine Health District in Italy (5 males, 8 females, median age (Q1–Q3) 54.0 (42.0–67.5)) years participated in this study. The median (Q1–Q3) length of service in primary care was 24.0 (14.5–40.5) years. Each participating physician extracted through the clinical record management software a complete list of patients with a confirmed diagnosis of DM2. Patients with type 1 diabetes and gestational diabetes were excluded. The patient grid/record was divided into nine parts.

GP’s details: surname, name, identification code, gender, age, work experience.

Patient’s details: anonymized code, gender, age, ethnicity (Caucasian/Other), body mass index (kg/m²), menopause (absent/present/not applicable), smoking (no/past/current), alcohol consumption (no/below/above 40–30 gr/day for males/females), education (elementary–middle/high school/university), work/economic capacity (absent/precarious/good), family composition (alone/with spouse/with others), physical activity (absent/mild/adequate).

Concurrent pathologies (present/absent): cardiovascular (no hypertension or ischemic heart disease), respiratory, upper digestive tract, liver/biliary tract, hematological, rheumatological, renal (no diabetic nephropathy), neoplastic, thyroid, neurological (no diabetic neuropathy or vascular cerebropathy), psychiatric (anxiety/depression/psychosis), frailty (CSHA score).

Diabetic disease: age at diagnosis, disease duration.

Arterial hypertension and micro/macro-vascular complications five years before study entry: arterial hypertension (no/1st–2nd/3rd stage), diabetic nephropathy (no/mild–moderate/severe), peripheral neuropathy (no/mild–moderate/severe), diabetic retinopathy (no/mild–moderate/severe), ischemic heart disease (no/mild–moderate/severe), peripheral vascular disease (no/mild–moderate/severe), abdominal aortic aneurysm (no/mild–moderate/severe), cerebrovascular disease (no/mild–moderate/severe).

As above but at the time of study entry.

Laboratory tests/blood pressure/medical therapy 24–36 months before study entry: recorded, if present, data for blood glucose (mg/dL), glycated hemoglobin (%), number of glycated hemoglobin determinations, serum creatinine (mg/dL), glomerular filtration rate (mL/min), number of creatinine determinations, microalbuminuria (mg/L), number of microalbuminuria determinations, total/HDL/LDL cholesterol/triglycerides (mg/dL), number of LDL determinations, systolic/diastolic blood pressure (mmHg), diabetes therapy (diet/metformin/other oral antidiabetics/basal insulin/intensive insulin), arterial hypertension therapy (diet/monotherapy/dual therapy/triple or more therapy), hypercholesterolemia therapy (diet/statin/statin plus ezetimibe).

As above but 12–24 months before study entry.

As above but 0–12 months before study entry and antithrombotic therapy (absent/antiaggregant/anticoagulant).

Regarding the five-year retrospective study, the following clarifications are provided:

- For each parameter, 0 was assigned in case of absence, 1 in case of mild–moderate, 2 in case of severe disease.
- Arterial hypertension was graded with score 1 assigned to first–second and 2 to third stage.
- Diabetic nephropathy was classified as mild–moderate in the presence of proteinuria ≤3.0 g/d with modest reduction in renal function (GFR > 30 mL/min) and severe in the presence of severe renal insufficiency (≤30 mL/min) and/or proteinuria >3.0 g/d.
- Diabetic peripheral neuropathy was considered mild–moderate in case of modest symptomatology with EMG confirmation, severe in case of invalidating symptomatology and EMG finding corresponding to clinical severity.
- Diabetic retinopathy was classified as mild–moderate in case of compatible instrumental finding in the absence of significant vision loss, severe when there was significant vision loss.
- Ischemic heart disease was considered mild–moderate when clinical and instrumental elements were compatible with ischemia without hearth necrosis, severe in the presence of previous hearth necrosis (NSTEMI/STEMI).
- Peripheral vascular disease was considered mild–moderate in presence of “intermittent claudication” and radiographic diagnostics compatible with perfusion deficit, severe in presence of previous revascularization interventions of various types.
- Abdominal aortic aneurysm was considered mild–moderate in absence of characteristics indicating vascular treatment, severe in remaining cases.
- Cerebral vascular disease was considered mild–moderate in presence of carotid stenosis indicating surgical intervention and/or previous history of TIA, severe in presence of previous ischemic/hemorrhagic stroke.

Regarding the three-year retrospective study, the following clarifications are provided:

- In case of multiple results present in a time interval, the average of values was calculated.
- For glycated hemoglobin, serum creatinine, microalbuminuria, lipids, the number of determinations in the time interval also had to be reported (0 = absence of values).
- Regarding arterial hypertension, the presence or absence of measurement in the considered time interval was indicated, 0 indicating absence and 1 presence of one or more measurements.

The study was performed in accordance with the principles included in the Declaration of Helsinki.

## 3. Statistical Analysis

Statistical analysis was performed using the following software: BMDP, MEDCALC, STATA 15. Categorical variables were expressed as absolute numbers and percentages, continuous variables with normal distribution as mean (standard deviation) and with non-normal distribution as median (interquartile range). Association between categorical variables was performed using chi-square test (for linear trend when appropriate). In the case of paired data for categorical variables, McNemar’s symmetry test was used. The difference between groups for continuous variables with normal distribution was investigated using one-way analysis of variance. Correlation between continuous variables was investigated using Pearson’s linear correlation coefficient. Analysis of covariance, considering age as co-variate, was performed to adjust variance analysis data corrected for patient age. Repeated measures analysis of variance was performed to evaluate changes in values of a variable over time, between groups and the interaction between these two parameters. Stepwise logistic regression was performed to identify independent predictive factors of a binary categorical variable; in the case of ternary categorical variables, stepwise multinomial regression was used. Negative binomial regression was performed to identify both continuous and categorical variables associated with a dependent variable with excess zeros. Operating curves were constructed to evaluate and compare the discriminative value of some parameters.

## 4. Results

### 4.1. Population

In total, 1169 patients with DM2 were recruited. Table 1 reports the socio-demographic characteristics of the studied population. In 567 cases (48.5%), there was a history of previous or current smoking and in 126 (10.8%) the presence of significant (>30–40 gr/day) alcohol consumption. A strong association was found between male gender and history of past or current smoking (402/663 vs. 165/506, *p* < 0.0001) and between male gender and increasing alcohol consumption (no 244/619 vs. mild/moderate 312/424 vs. severe 107/126, *p* < 0.0001).

### 4.2. Comorbidities (Table 2)

In 148 (12.7%) patients, no comorbidities were detected. In 249 (21.3%), one comorbidity was present; in 266 (22.8%) two, in 230 (19.7%) three, in 147 (12.6%) four, and in 129 (11.0%) five or more additional diseases, of those recorded, were observed. Cardiovascular disease was the illness most frequently (30.2%) associated with DM2, even excluding arterial hypertension and diseases related to diabetic micro/macroangiopathy. The second most frequent disease was thyroid pathology (28.4%), followed by diseases of the upper digestive tract (27.9%). Finally, regarding frailty, only 3.9% of subjects were very well performing, and the vast majority (N = 913, 78.1%) did not present an important frailty. Otherwise, significant frailty was found in 210 (18.0%) patients. A higher prevalence of thyroid, psychiatric, dyspeptic, as well as neurological pathology not strictly related to diabetes emerges in females. Furthermore, women were found to have more frequently a severe grade of frailty than men. Regarding age, a strong association was observed between more advanced age and higher frequency of cardiovascular, hematological, nephrological (excluding diabetic nephropathy), neoplastic, and neurological pathology not strictly related to diabetes. Finally, a strong association between age and entity of frailty was found.

**Table 2:** Occurrence of co-morbidity in the studied population (N = 1169). Categorical variables are presented as absolute number and percentage (%), continuous variables as median interquartile range (Q1–Q3). For any disease, total values and percentage in relationship with gender (males/females) and age (≤70/71–80/≥81 years) are presented. Statistical analysis was performed by means of chi-square test (for linear trend when appropriate).

Disease	Total N = 1169	Males N = 663	Females N = 506	*p*	≤70 N = 425	71–80 N = 403	≥81 N = 341	*p*
Cardiovascular *	353	30.5%	29.8%	0.817	18.1%	30.5%	44.9%	<0.0001
Respiratory	237	22.9%	16.8%	0.010	16.7%	24.8%	19.4%	0.278
Esophagus–gastric	326	22.9%	34.4%	<0.0001	24.0%	28.3%	32.3%	0.011
Hepato-biliary	300	25.9%	25.3%	0.802	25.4%	27.0%	24.3%	0.775
Hematologic	211	18.9%	17.0%	0.413	9.4%	18.9%	27.9%	<0.0001
Rheumatologic	110	8.0%	11.3%	0.058	6.1%	12.2%	10.3%	0.036
Nephrology **	159	14.8%	12.1%	0.178	6.8%	16.4%	18.8%	<0.0001
Neoplastic	225	19.0%	19.6%	0.810	11.8%	22.1%	25.2%	<0.0001
Thyroid	332	17.2%	43.1%	<0.0001	25.6%	29.8%	30.2%	0.151
Neurology ***	200	14.5%	20.6%	0.006	6.4%	19.9%	27.3%	<0.0001
Psychiatry Anxiety Depression Psychosis	318	16.6%	41.1%	<0.0001	26.6%	26.6%	28.7%	0.522
	150	34.0%	66.0%	0.922	38.0%	36.0%	26.0%	0.018
	155	35.5%	64.5%		30.3%	32.3%	37.4%	
	13	30.8%	69.2%		69.2%	23.1%	7.7%	
^▲^ CSHA > 3	388	27.3%	40.9%	<0.0001	12.9%	27.3%	65.4%	<0.0001
^▲^ CSHA/Median = 3/Q1–Q3 = 2–4								

* No hypertension or ischemic cardiopathy, ** no diabetic nephropathy, *** no diabetic neuropathy or diabetic cerebral vasculopathy, ^▲^ Canadian Society of Health and Aging frailty scale.

### 4.3. Organ Damage Progression

**Figure 1:**
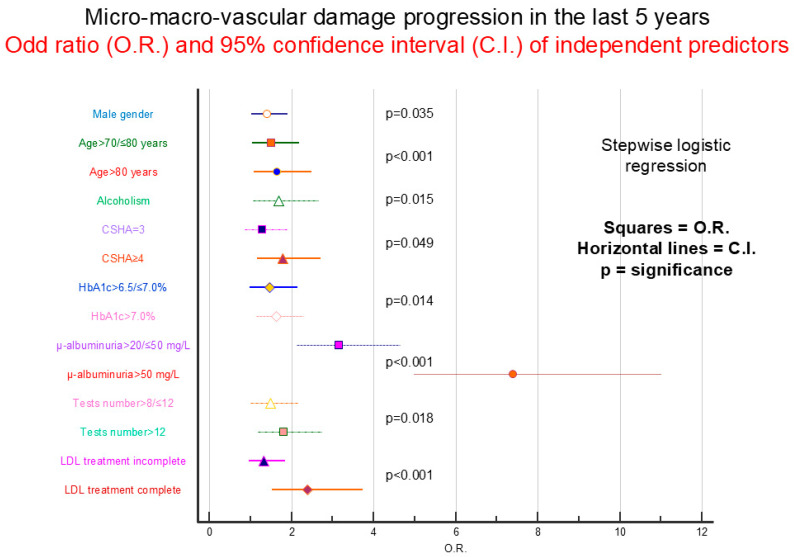
Stepwise logistic regression analysis performed to identify independent predictors of micro/macroangiopathy progression (sum of all angiopathy components) starting from five years before to the entry into the study. Laboratory parameters were expressed as mean values obtained from the last three years. The number of biochemical tests represent the sum of the following: glycated hemoglobin, serum creatinine, microalbuminuria, and LDL cholesterol determinations performed during the last three years. Treatment with lowering cholesterol agents was checked annually and expressed as the sum of the last three years.

Table 3 reports the frequency of finding arterial hypertension and various components of diabetic micro/macroangiopathy. Of each component, the presence and gradation of the complication is reported, both at the time of study entry and what was present five years before. It is noted how an increase in frequency of all various complications can be found in the five intervening years considered. Specifically, 314 (26.9%) patients presented data of progression of diabetic micro/macroangiopathy in the last five years. Table 4 reports the association between demographic, environmental, clinical, and laboratory variables and the frequency of diabetic micro/macroangiopathy progression. A strong positive association is observed between frequency of damage progression and male gender, more advanced age, disease duration, smoking history, significant alcohol consumption, frailty, history of arterial hypertension, higher values of glycated hemoglobin and microalbuminuria, greater number of laboratory tests performed in the last three years and extent of lipid-lowering therapy practiced in the same period. Conversely, there was a strong negative association with adequate physical activity, GFR values, and LDL cholesterol levels in the last three years. Figure 1 reports the results of stepwise logistic regression. All variables present in Table 4 were included in the analysis in addition to years at diagnosis and values of serum creatinine, cholesterol, and triglycerides. The presence (>20 mg/L) and extent (>50 mg/L) of microalbuminuria was the most important independent variable in predicting micro/macro-vascular damage progression. In this regard, it should be noted that only one determination of microalbuminuria in the three years prior to study entry was found in 34.6% of patients, a percentage significantly higher than that found for glycated hemoglobin (13.1%, *p* < 0.0001), creatinine (8.9%, *p* < 0.0001), and lipid profile (18.1%, *p* < 0.0001). However, a significant association was detected between the occurrence of organ damage progression and number of microalbuminuria determinations (one 98/404 vs. two 85/339 vs. three or more 131/426 *p* = 0.033). Furthermore, damage progression was also associated with more sustained lipid-lowering therapy. Repeated measures analysis of variance was performed considering the total score related to micro/macro-vascular damage plus any presence and gradation of arterial hypertension. The mean score (standard deviation) is reported at time −5 years vs. time 0 (study entry) classifying patients based on gender [males 1.52 (1.48) vs. 2.08 (1.73), females 1.29 (1.16) vs. 1.69 (1.34)], age in years [>75 1.76 (1.43) vs. 2.33 (1.61), ≤75 1.12 (1.21) vs. 1.54 (1.47)], smoking history [present 1.67 (1.56) vs. 2.22 (1.78), absent 1.19 (1.08) vs. 1.62 (1.31)], and high alcohol consumption [present: 1.71 (1.47) vs. 2.28 (1.82), absent 1.39 (1.34) vs. 1.87 (1.55)]. The score always increases over time (*p* < 0.0001) and was higher in males (*p* = 0.0002), in subjects > 75 years (*p* < 0.0001), in patients with smoking history (*p* < 0.0001), and in those who had excessive alcohol consumption (*p* = 0.007). The increase in score over time was greater in males compared to females (*p* = 0.0005), in older subjects compared to younger ones (*p* = 0.0007), and in smokers compared to non-smokers (*p* = 0.009). Negative binomial regression was performed between all demographic, laboratory, and clinical variables considered and progression of micro/macro-vascular damage, including arterial hypertension, calculated from five years before entry into the study. Damage progression was independently associated with male sex (*p* = 0.005), presence of non-diabetic neurological pathology (*p* < 0.001), microalbuminuria values (0.019), total number of laboratory tests performed (*p* = 0.025), and extent of antihypertensive therapy (*p* = 0.017) in the last three years. The association was negative with the presence of better economic resources (*p* = 0.035) and with higher LDL values (*p* = 0.047).

### 4.4. Gender/Age

Table 5 reports the analysis of variance of demographic/clinical and laboratory parameters dividing patients based on gender. It emerges that females are older, with a more advanced age at diagnosis and with greater frailty. Regarding laboratory parameters (mean values of last three years), higher creatinine and glomerular filtration rate are reported in males, while total cholesterol, HDL, and LDL were found to be higher in females. A strong correlation was also observed between patient age at study entry and most variables: positive with CSHA values, creatinine, and HDL cholesterol and negative with BMI, blood glucose, glycated hemoglobin, glomerular filtration rate, total triglycerides, and LDL cholesterol. Covariance analysis of clinical and laboratory parameters, adjusting data for age, confirmed and reinforced variance analysis data. These analyses were repeated considering only patients who had not previously presented important macro-vascular events (N = 983), obtaining a faithful replication of results.

### 4.5. LDL Cholesterol Variations

**Figure 2:**
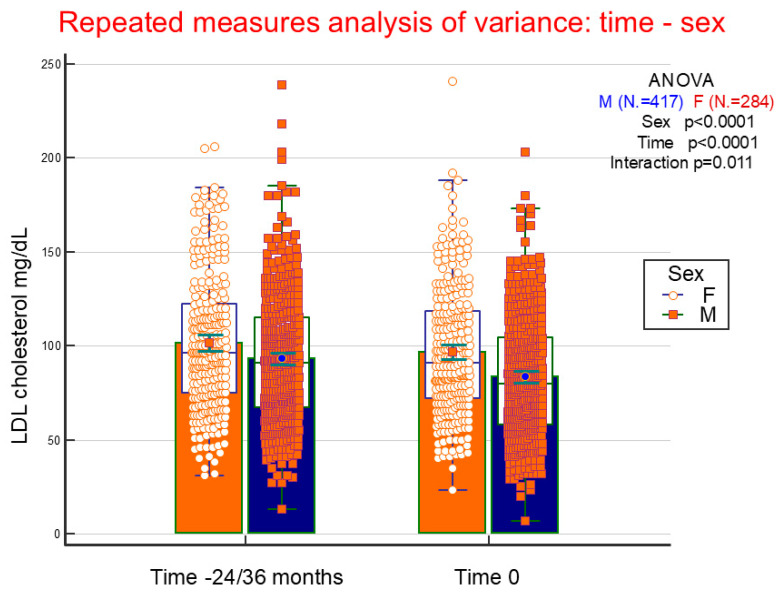
Repeated measures analysis of variance of serum LDL cholesterol in the 701 patients with the value at the entry into the study (0) and three years before (-24/36 months). Within groups refers to the variations from the first to the second time point, between groups refers variations between males and females, and interaction verifies whether variations during time differ significantly in relationship to gender (males and females). Statistical analysis was performed using a logarithmic transformation of the data.

Stepwise multinomial regression was performed to highlight independently predictive variables of progressively decreasing values (71–130 and ≤70 mg/dL compared to >130 mg/dL) of LDL cholesterol (Table 6). Mean LDL cholesterol values ≤ 70 mg/dL are significantly associated with male gender, more advanced age, longer disease duration, smoking history, not living alone, history of arterial hypertension, as well as lipid-lowering therapy. Conversely, excessive alcohol consumption appears to be associated with higher LDL values. Figure 2 shows changes in LDL cholesterol values, from 24/36 months before to time of study entry, in 701 patients (417 males and 284 females) who possessed both determinations, dividing them based on gender. It appears evident how LDL cholesterol is higher in females, decreases over time and this occurs more significantly in males.

### 4.6. Advanced Age, Male Gender, Smoking History, and Treatment

An association was detected between male patients aged above 70 years with a smoking history (N. = 257, 22.0%), in comparison to the remaining patients, and the intensity of DM2-related therapies. This applied to glucose-lowering treatment (low intensive 54/292 vs. intermediate 86/431 vs. high 117/446, *p* = 0.008), treatment of hypertension (low intensive 44/310 vs. intermediate 143/604 vs. high 70/255, *p* < 0.001), and cholesterol-lowering treatment (low intensive 86/491 vs. intermediate 129/528 vs. high 42/150, *p* = 0.001). Being never treated with statin (N. = 491, 42.0%) was observed in 85/165 females aged ≤ 70 years, 125/260 males aged ≤ 70 years, 134/341 females aged > 70 years, 147/403 males aged > 70 years; *p* < 0.001. No statin treatment occurred in 218/567 patients with smoking history vs. 273/602 never smokers (*p* = 0.017).

### 4.7. Current and 10-Year Cardiovascular Risk

**Figure 3:**
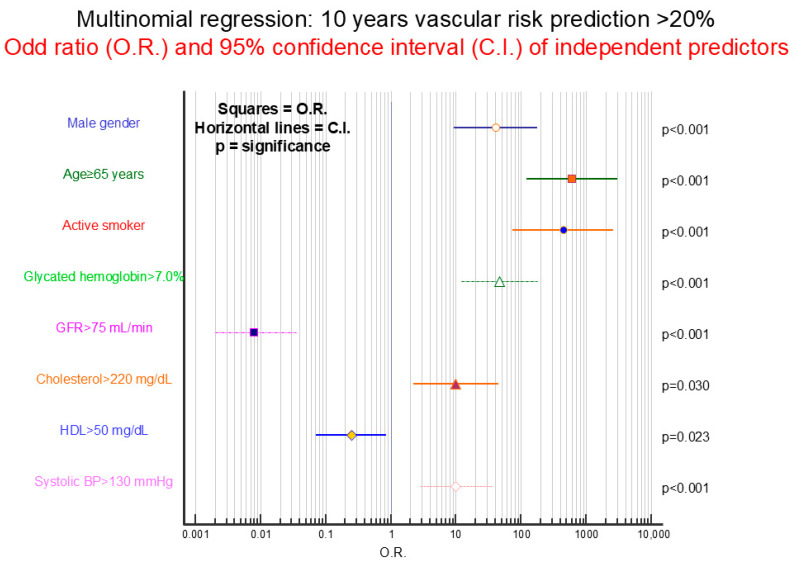
Stepwise multinomial regression analysis performed in the 393 patients aged < 70 years to identify, among SCORE2 variables, those more predictive of an 11–20% or >20% ten-year vascular risk in comparison to a ≤10% value. The figure shows the results of the >20% ten-year vascular risk. The mean values of the last three years were used for laboratory parameters and arterial blood pressure measurements.

**Figure 4:**
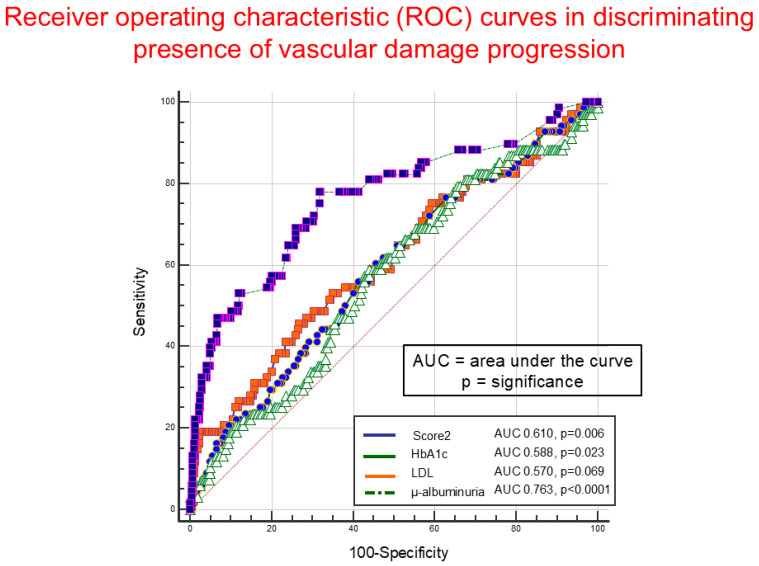
Receiver operating characteristic (ROC) curves of microalbuminuria, LDL cholesterol, glycated hemoglobin, and SCORE2 in discriminating, among the 393 subjects aging < 70 years, those with presence of micro/macro-vasculopathy progression (N = 68, 17.3%) evaluated at the entry into the study. The figure shows the area under the curve (AUC) values and their statistical significance.

Ten-year cardiovascular risk was calculated using the specific application recently licensed by the European Society of Cardiology (SCORE2-DIABETES). Risk was calculated in 393 younger subjects (243 males and 150 females) with age included between 40 and 69 years. Based on the calculation, risk of cardiovascular events at 10 years was estimated at ≤10% in 126 subjects, 11–20% in 225, and >20% in 42. Stepwise multinomial regression was performed to highlight, among score variables, those predictive of 10-year risk of 11–20% or >20% compared to ≤10%. Figure 3 shows how >20% risk at 10 years is predicted mainly by more advanced age, being an active smoker, as well as male gender and higher glycated hemoglobin values. Blood pressure and cholesterol values seem to play a less important role. Good renal function and higher HDL cholesterol values appear to play a protective role. Figure 4 reports operating curves of SCORE2, glycated hemoglobin, LDL cholesterol, and microalbuminuria in identifying, in these 393 subjects, those in whom progression of micro/macro-vascular organ damage was found (N = 68, 17.3%) at time of study entry. It is observed that microalbuminuria is clearly more performant than other parameters, with an area under the curve significantly greater than SCORE2 (*p* = 0.001), glycated hemoglobin (*p* = 0.0005), and LDL cholesterol (*p* = 0.0001).

## 5. Discussion

In this study, 1169 subjects were recruited from a population of 18,100 patients, with a disease prevalence of 6.46%. Male gender and an age > 70 years was recorded in 56.7% and in 63.6% of cases, respectively. Our data perfectly aligned with the official data reported for the adult Italian Friuli–Venezia Giulia Region (EpiCentro 2020). Our patients were generally overweight, similar to national data reporting presence of overweight in 71% of patients. Regarding smoking, considering smokers and former smokers cumulatively, we obtained a prevalence overlapping with that detected by the PASSI study (2022) performed in the general population of Friuli–Venezia Giulia. Regarding alcohol consumption, our data showed a significant consumption in 10.8% of cases, which was lower than reported by the PASSI study (2022) conducted in our region. However, it could be difficult to precisely categorize the degree of alcohol consumption and other studies [17] reported significant alcohol consumption in <10% of patients. Only a small proportion of patients engaged in adequate physical activity compared to recommendations; however, in this case series, a high proportion of patients were elderly. Regarding environmental and independently modifiable risk factors, our diabetic patients had a profile similar to that of the general population and were therefore not at target for their condition [18]. Thus, the role of the GP in this context could assume enormous relevance.

DM2 is characterized by the presence of micro- and macro-vascular complications specific to the disease [2,13,19,20]. Frequently, clinical conditions called “concordant” with diabetic disease [21,22], such atrial fibrillation, other rhythm disturbances, and heart failure, were also present in these patients. However, due to the increased patient survival, diabetes manifested in association with non-vascular so-called “discordant” pathologies [21,23]. We found respiratory diseases, such as sleep apnea, and hepatobiliary conditions as hepatic steatosis [24]. The increased life expectancy of these patients accounted for the high rates of neoplastic disease and dementia recorded [25,26]. Therefore, frailty, specific or not to diabetic disease, strongly correlated with age and female gender.

The most frequently observable cardiovascular pathology in patients with DM2 is arterial hypertension, and this was confirmed in the present study [27,28]. At the time of enrollment, 78.4% of patients presented with this pathology, in most cases already present 5 years before. Among microvascular complications, we observed a higher prevalence of diabetic nephropathy, while the prevalence of diabetic retinopathy was modest at 6.67%, lower than that reported in other Italian case series [29,30]. The data could represent an underestimate of the real situation, partly related to the difficulty in having type 2 diabetic patients undergo fundus examinations in times of reduced National Health System resources. Our data on the prevalence of macro-vascular complications in type 2 diabetics were entirely overlapping with national data available on the Ministry of Health website (EpiCentro 2020). Among these, the most frequent was found to be ischemic heart disease followed by cerebral vasculopathy. Regarding ischemic heart disease, it should be emphasized that in patients with DM2, infarction disease may be more frequently found compared to other less severe forms of disease [31]. It is known that for various pathophysiological reasons, particularly due to autonomic neuropathy [32], the onset of coronary disease in these patients may more frequently represent a serious event compared to other categories of patients [33].

Several factors are known to possess a predictive role in the evolution of vascular damage in patients with DM2 [30,34]. Certainly, smoking history (past or current) [35], lack of physical activity [12,36], presence of arterial hypertension, poorer control of glycated hemoglobin values, and higher levels of creatinine, microalbuminuria, and cholesterol have been repeatedly associated with progression of vascular damage [4,5,30]. In the present work, the detection and extent of microalbuminuria appeared to be strongly associated with the presence of damage progression [37]. In the literature, the relationship between alcohol consumption and vascular damage progression remained uncertain, and proved only for high alcohol consumption [38]. Indeed, in this study, the relationship between vascular damage progression and alcohol was fully manifested for elevated consumption of alcoholic beverages. A further less-shared observation concerned the association between frailty and vascular damage progression. The apparently paradoxical inverse association between LDL cholesterol values and vascular damage progression left us puzzled. In fact, decreasing LDL values, induced by a greater adoption of lipid-lowering therapy, were associated with significantly increased vascular damage progression. At the multivariate analysis, microalbuminuria and the extent of lipid-lowering therapy were found to be the major predictors of damage progression. It might be hypothesized that in this condition, control of cholesterol values is achieved when renal damage is already established. It may be suggested that GPs adjust lipid-lowering therapy not evaluating an “a priori” risk but, on the contrary, considering the vascular events that have occurred during the patient’s clinical history. Therefore, this therapy will be generally intensified over time and with the worsening of the patient’s clinical conditions. Certainly, the crucial role of microalbuminuria in predicting organ damage progression in our series could be lowered by the fact that this test was performed less frequently than other laboratory determinations useful to monitor DM2. Nevertheless, it must be emphasized that in patients with vascular damage progression, a more frequent determination of microalbuminuria was performed in comparison to patients without damage progression. Finally, the emerging role of co-morbidities should not be neglected, even if not strictly related to diabetic disease [22].

Although the relationship between more advanced age and the presence of more severe micro/macro-vascular damage is expected, it might not be linear. In our study, we observed a relationship not only with damage but also with the extent of micro/macro-vascular damage progression: the greater the age, the greater the extent of progression appears to be. Indeed, the literature indicates that renal damage progression is not linear and worsens in the presence of a longer disease history [39,40].

The relationship between gender and vascular damage progression in patients with DM2 remains a controversial topic [29,41,42,43], as contrasting elements intersect. In the general population, male gender is associated with a higher cardiovascular risk than female. In contrast, recent studies would have highlighted how in women the relative cardiovascular risk specifically attributable to the presence of diabetes is greater than that observable in men [44]. Clear evidence indicates that sex-related and gender-related factors interact in generating differences in cardiovascular outcomes in women and men and might even have opposite effects on clinical manifestations and outcomes [45]. The influence of biological sex on cardiovascular manifestations frequently favors females, such as the relative protection from obstructive coronary artery disease in premenopausal females. Conversely, gender-related factors, including a higher prevalence of smoking and of heavy alcohol consumption in males, observed in the present study, could more adversely affect men than women. Accordingly, the more-frequent vascular damage progression recorded in males supports the observations and reports that the absolute cardiovascular risk of the diabetic patient in Italy is greater in men compared to women [16].

Compared to works conducted in the specialist setting, studies conducted in primary care demonstrated that clear benefits in controlling diabetic complications are obtainable only in patients with poor disease control [46] and how comorbidities and socioeconomic status are strongly predictive of worse outcomes [47]. Primary care is characterized by an undifferentiated access, continuity over time and space, the search for a “therapeutic alliance” with the patient through dialogue, and using a holistic approach. These specific aspects may represent limitations with respect to the treatment of a specific disease such as DM2 when handled in the context of the globality of patient health. Nevertheless, the role of the GP appears to remain important [48]. GPs consider the female diabetic patient at lower clinical risk compared to males [49], probably because the disease in women appears later when other comorbidities are present. Therefore, the achievement of controlled lipid profile is perceived as less cogent for females [50,51].

The analysis of SCORE2 cardiovascular risk components in our younger patients clearly demonstrated how age, male gender, and active smoker status, as well as glycated hemoglobin values, constituted much stronger risk predictor elements than the cholesterol value itself. Smoking history is exemplary as a predominantly male risk factor and to be associated with more vigorous lipid-lowering therapy, while this is not apparent for excessive alcohol consumption [52]. Finally, in our study, living alone, more frequent in women, has proven to be an independent predictive factor of higher LDL values.

Although the results presented in this study could be valuable in the context of the Italian population, they cannot be simply extrapolated to other populations [53]. As an example, in Asian patients, diabetes develops at a younger age and is characterized by early cell dysfunction in the setting of insulin resistance, which may require early insulin treatment. East Asian patients with DM2 have a higher risk of developing renal complications than Europeans and, with regard to cardiovascular complications, a predisposition for developing strokes [54]. Moreover, four regions have been identified in Europe possessing different levels of cardiovascular risk, from low to very high risk, with Italy being included among moderate risk regions [55]. This fact precludes generalizations of our results to other populations, particularly what concerns the effect of male gender on the progression of cardiovascular damage.

This study is retrospective; surely prospective studies are needed to accurately verify the long-term effects of predictive factors of vascular damage progression in DM2. Nevertheless, this study provides several suggestions particularly useful in the primary care context. First, GPs should greatly encourage patients to stop smoking and excess drinking, since smoking, particularly, increases the risk of vascular damage progression. This intervention must be performed early and in every patient. Secondly, GPs should include microalbuminuria determination at least annually early during the history of DM2, especially in patients with other cardiovascular risk factors, such as male gender, smoking history, and presence of hypertension. Thirdly, in patients presenting these factors, GPs should consider the option to anticipate and intensify lipid-lowering therapy in relationship with the other specific interventions. Unfortunately, younger male who are smokers are generally a category of patients that is not very adherent [56,57].

In conclusion, older age and male gender were strongly associated with the presence and extent of vascular damage progression, as well as a history of smoking. The GP has a differentiated approach in evaluating the risk factors (particularly lipid profile) of damage progression. The GP adopts an intensive therapeutic approach in males, the elderly, and smokers. Microalbuminuria was the marker most strongly associated with the presence of vascular damage progression.

## Figures and Tables

**Table 1:** Demographic and clinical characteristics of the studied population affected by DM2 (N = 1169). Categorical variables are presented as absolute number (N) and percentage (%). Continuous variables are presented as mean (ME) and standard deviation (SD).

Male Gender N (%)		663 (56.7)
Age Years ME (SD)		73.5 (11.0)
Age in years at diagnosis ME (SD)		61.6 (11.6)
Caucasian ethnicity N (%)		1145 (97.9)
Body mass index (Kg/m^2^) ME (SD)		28.4 (5.2)
Menopause	Not applicable N (%)	663 (56.7)
	Absent N (%)	14 (1.2)
	Present N (%)	492 (42.1)
Smoking	Never N (%)	602 (51.5)
	Past N (%)	443 (37.9)
	Current N (%)	124 (10.6)
Alcohol	Never N (%)	619 (52.9)
	Mild * N (%)	424 (36.3)
	Severe * N (%)	126 (10.8)
School	Elementary/middle N (%)	691 (59.1)
	Primary N (%)	382 (32.7)
	Graduate N (%)	96 (8.2)
Work/income	Absent N (%)	48 (4.1)
	Low N (%)	168 (14.4)
	Sufficient N (%)	953 (81.5)
Family support	Alone N (%)	269 (23.0)
	Married N (%)	703 (60.1)
	With others N (%)	197 (16.9)
Physical activity	Absent N (%)	556 (47.6)
	Low N (%)	486 (41.6)
	Regular N (%)	127 (10.9)

* Alcohol consumption ≤ 30–40 g/day, for females and males, respectively, was considered mild whereas above these thresholds was considered severe.

**Table 3:** Presence and entity, in the studied population (N = 1169), of arterial hypertension and diabetic micro/macroangiopathy, recorded five years before and at the entry into the study. Statistical analysis was performed by means of MCNEMAR test for paired data.

		Five Years Before			At the Entry			
		No N (%)	Mild/Moderate N (%)	Severe N (%)	No N (%)	Mild/Moderate N (%)	Severe N (%)	*p*
	Arterial hypertension	332 (28.4)	697 (59.6)	140 (12.0)	252 (21.6)	702 (60.1)	215 (18.4)	<0.0001
Microangiopathy	Diabetic nephropathy	1035 (88.5)	126 (10.8)	8 (0.7)	909 (77.8)	232 (19.8)	28 (2.4)	<0.0001
	Diabetic neuropathy	1126 (96.3)	38 (3.3)	5 (0.4)	1087 (93.0)	72 (6.2)	10 (0.9)	<0.0001
	Diabetic retinopathy	1115 (95.4)	48 (4.1)	6 (0.5)	1091 (93.3)	69 (5.9)	9 (0.8)	<0.0001
Macroangiopathy	Ischemic cardiopathy	1046 (89.5)	28 (2.4)	95 (8.1)	997 (85.3)	48 (4.1)	124 (10.6)	<0.0001
	Peripheral vasculopathy	1103 (94.4)	53 (4.5)	13 (1.1)	1084 (92.7)	66 (5.6)	19 (1.6)	<0.0001
	Abdominal aortic aneurism	1157 (99.0)	8 (0.7)	4 (0.3)	1146 (98.0)	19 (1.6)	4 (0.3)	0.0009
	Cerebral vasculopathy	1081 (92.5)	55 (4.7)	33 (2.8)	1013 (86.7)	99 (8.5)	57 (4.9)	<0.0001
Microangiopathy *		977 (83.6)	151 (12.9)	41 (3.5)	825 (70.6)	255 (21.8)	89 (7.6)	<0.0001
Macroangiopathy *		924 (79.0)	102 (8.7)	143 (12.2)	819 (70.1)	144 (12.3)	206 (17.6)	<0.0001
Micro/macroangiopathy *		803 (68.7)	167 (14.3)	199 (17.0)	618 (52.9)	247 (21.1)	304 (26.0)	<0.0001

Scores: 0 = absence of disease, 1 = mild/moderate disease, 2 = severe disease. * Sum of single micro/macroangiopathy components.

**Table 4:** Variables associated with micro/macroangiopathy progression in the studied population (N = 1169). Progression was present when the sum of micro/macroangiopathy components at the entry into the study was superior to that found five years before. Statistical analysis was performed by means of chi-square test (for linear trend when appropriate).

Presence and Percentage of Micro/Macroangiopathy Progression					*p*
Gender	Female N = 109 (21.5)		Male N = 205 (30.9)		0.0003
Age years	≤70 N = 75 18%	71–80 N = 120 30%		≥81 N = 119 35%	<0.0001
Years of diabetes	≤10 N = 109 21%	11–15 N = 80 28%		≥16 N = 125 36%	<0.0001
BMI kg/m^2^	≤25 N = 92 31%	26–30 N = 119 24%		≥31 N = 103 27%	0.337
Smoking	Absent N = 142 24%		Present N = 172 30%		0.009
Heavy alcohol	Absent N = 267 26%		Present N = 47 37%		0.005
School	Middle N = 190 28%	Diploma N = 97 25%		Degree N = 27 28%	0.744
Income	Poor N = 16 33%	Modest N = 45 27%		Good N = 253 27%	0.428
Family	Alone N = 75 28%	Spouse N = 184 26%		Others N = 55 28%	0.945
Physical activity	No N = 173 31%	Light N = 114 24%		Good N = 27 21%	0.002
CSHA	≤2 N = 64 18%	3 N = 111 27%		≥4 N = 139 36%	<0.0001
Hypertension *	Absent N = 66 20%		Present N = 248 30%		0.0007
HbA1c % **	≤6.5 N = 99 21%	6.6–7.0 N = 78 28%		>7.0 N = 137 33%	<0.0001
GFR mL/min **	≤45 N = 57 48%	46–60 N = 64 33%		>60 N = 193 23%	<0.0001
µALBU mg/L **	≤20 N = 146 17%	21–50 N = 66 44%		>50 N = 102 65%	<0.0001
HDL mg/dL **	≤40 N = 52 29%	41–60 N = 172 27%		>60 N = 90 25%	0.300
LDL mg/dL **	≤70 N = 115 38%	71–130 N = 170 24%		>130 N = 29 18%	<0.0001
Tests number ***	≤8 N = 57 18%	9–12 N = 152 27%		>12 N = 105 35%	<0.0001
LDL therapy ^	No N = 98 20%	Partial N = 157 30%		Regular N = 59 39%	<0.0001

BMI: body mass index; CSHA: Canadian Society of Health and Aging frailty scale; HbA1c: glycated hemoglobin; GFR: glomerular filtration rate; μALBU: microalbuminuria; HDL: high-density lipoprotein; LDL: low-density lipoprotein. * Five years before the entry into the study. ** Laboratory parameters were expressed as mean values obtained from the last three years. *** The number of biochemical tests represent the sum of the following: glycated hemoglobin, serum creatinine, microalbuminuria, and LDL cholesterol determinations performed during the last three years. ^ Treatment with cholesterol-lowering agents was checked annually and expressed as the sum of the last three years.

**Table 5:** Demographic and clinical variables in the studied population (N = 1169) divided in relationship with patients’ gender (males/females). Data are presented as mean (standard deviation: SD). Statistical analysis was performed using one-way analysis of variance (ANOVA) (left side). The correlation between variables and patient’s age was performed using the Pearson test. Covariance analysis was performed considering age as covariate (ANCOVA) (right side). Laboratory results represent the means of the last three years.

	Males	N = 663	Females	N = 506	ANOVA				
	Mean	SD	Mean	SD	*p*				
Age in years	72.5	10.6	74.8	11.4	0.0003				
Years at diagnosis	60.4	11.0	63.2	12.2	<0.0001	**Correlation with age**			**ANCOVA**
Years with the disease	12.1	7.8	11.6	7.6	0.319	Coefficient	F	*p*	*p*
BMI (kg/m^2^)	28.4	4.8	28.5	5.6	0.867	−0.088	42.8	<0.0001	0.386
CSHA	3.16	1.44	3.56	1.52	<0.0001	0.064	336	<0.0001	0.001
Mean serum glucose (mg/dL)	134	33	130	33	0.034	−0.267	9.42	0.002	0.070
Mean glycated hemoglobin (%)	6.92	1.04	6.88	0.95	0.577	−0.009	11.1	0.0009	0.834
Mean serum creatinine (mg/dL)	1.11	0.66	0.88	0.30	<0.0001	0.004	7.99	0.005	<0.001
Mean glomerular filtration rate (mL/min)	75.2	21.1	71.2	22.2	0.002	−0.977	383	<0.0001	0.120
Mean cholesterol (mg/dL)	165	35	183	37	<0.0001	−0.444	20.6	<0.0001	<0.001
Mean HDL cholesterol (mg/dL)	51	13	58	15	<0.0001	0.117	9.22	0.002	<0.001
Mean triglycerides (mg/dL)	126	59	128	54	0.546	−0.785	27.7	<0.0001	0.240
Mean LDL cholesterol (mg/dL)	89	31	99	33	<0.0001	−0.402	22.9	<0.0001	<0.001

BMI: body mass index; CSHA: Canadian Society of Health and Aging frailty scale; HDL: high-density lipoprotein; LDL: low-density lipoprotein.

**Table 6:** Stepwise multinomial regression analysis performed to identify independent predictors of progressively decreasing LDL cholesterol values (71–130 and ≤70 mg/dL) in comparison to >130 mg/dL. For LDL cholesterol and creatinine, mean values during the last three years were considered.

	Mean Cholesterol Values 71–130 mg/dL			Mean Cholesterol Values ≤ 70 mg/dL			
	Coefficient	O.R.	95% C.I.	Coefficient	O.R.	95% C.I.	*p*
Male gender	0.683	2.0	1.3–3.0	1.174	3.2	1.9–5.4	<0.001
Age > 75 years	0.690	2.0	1.3–3.0	0.766	2.2	1.4–3.4	0.006
Smoking history	0.348	1.4	0.97–2.10	0.662	1.9	1.2–3.0	0.016
Alcohol consumption > 40 gr/day	−0.322	0.72	0.49–1.1	−0.769	0.46	0.29–0.74	0.008
Family components ≥ 2	0.447	1.6	1.1–2.3	0.704	2.0	1.2–3.3	0.019
Diabetes present from >10 years	−0.036	0.96	0.67–1.4	0.684	2.0	1.3–3.1	<0.001
Arterial hypertension from ≥5 years	−0.078	0.92	0.62–1.4	0.568	1.8	1.1–2.9	0.001
Mean creatinine > 0.90 mg/dL	−0.428	0.65	0.44–0.96	−0.082	0.92	0.58–1.5	0.017
Cholesterol treatment present	0.895	2.4	1.7–3.6	2.384	11	6.8–17	<0.001

O.R. = Odd ratio, C.I. = 95% confidence interval.

## Data Availability

The anonymized database could be available following motivated request.

## Abbreviations

Type two diabetes mellitus: DM2
General practitioner: GP
